# Multigene editing reveals that *MtCEP1*/*2*/*12* redundantly control lateral root and nodule number in *Medicago truncatula*

**DOI:** 10.1093/jxb/erab093

**Published:** 2021-02-26

**Authors:** Fugui Zhu, Qinyi Ye, Hong Chen, Jiangli Dong, Tao Wang

**Affiliations:** 1 State Key Laboratory of Agrobiotechnology, College of Grassland Sciences, China Agricultural University, Beijing, China; 2 State Key Laboratory of Agrobiotechnology, College of Biological Sciences, China Agricultural University, Beijing, China; 3 University of Warwick, UK

**Keywords:** CEP peptides, CRISPR/Cas9, lateral root, *Medicago truncatula*, multigene editing, symbiotic nodulation

## Abstract

The multimember *CEP* (*C-terminally Encoded Peptide*) gene family is a complex group that is involved in various physiological activities in plants. Previous studies demonstrated that *MtCEP1* and *MtCEP7* control lateral root formation or nodulation, but these studies were based only on gain of function or artificial miRNA (amiRNA)/RNAi approaches, never knockout mutants. Moreover, an efficient multigene editing toolkit is not currently available for *Medicago truncatula*. Our quantitative reverse transcription–PCR data showed that *MtCEP1*, *2*, *4*, *5*, *6*, *7*, *8*, *9*, *12*, and *13* were up-regulated under nitrogen starvation conditions and that *MtCEP1*, *2*, *7*, *9*, and *12* were induced by rhizobial inoculation. Treatment with synthetic MtCEP peptides of *MtCEP1*, *2*, *4*, *5*, *6*, *8*, and *12* repressed lateral root emergence and promoted nodulation in the R108 wild type but not in the *cra2* mutant. We optimized CRISPR/Cas9 [clustered regularly interspaced short palindromic repeats (CRISPR)/CRISPR-associated protein 9] genome editing system for *M. truncatula*, and thus created single mutants of *MtCEP1*, *2*, *4*, *6*, and *12* and the double mutants *Mtcep1/2C* and *Mtcep5/8C*; however, these mutants did not exhibit significant differences from R108. Furthermore, a triple mutant *Mtcep1/2/12C* and a quintuple mutant *Mtcep1/2/5/8/12C* were generated and exhibited more lateral roots and fewer nodules than R108. Overall, *MtCEP1*, *2*, and *12* were confirmed to be redundantly important in the control of lateral root number and nodulation. Moreover, the CRISPR/Cas9-based multigene editing protocol provides an additional tool for research on the model legume *M. truncatula*, which is highly efficient at multigene mutant generation.

## Teaser

Multiple *Mtcep* mutants generated using a modified CRISPR/Cas9 system reveal the redundant roles of *MtCEP1*, *2*, and *12* in controlling lateral root and nodule number in *Medicago truncatula*.

## Introduction

The root architecture system, which provides structural support and participates in the perception, absorption, allocation, and transport of nutrients, is essential for the survival of the whole plant (Motte *et al.*, 2019). The root system consists of primary and lateral roots, and its developmental responses are plastic to the heterogeneous soil environment, which enables immobile plants to adapt to the changing natural environment. Some specialized root-derived organs, such as nodules, where symbiosis is established between symbiotic bacteria and their host plants, also take part in the developmental process of the root system. This intricate system relies on various molecules that function in the coordination of different intercellular events (Takahashi *et al.*, 2019).

Small peptide hormones play vital roles in both growth-regulating and developmental processes and responding to environmental stress in the root system (Delay *et al.*, 2013b; Matsubayashi, 2014; Okamoto *et al.*, 2016). C-terminally encoded peptides (CEPs) are small, secreted peptides containing 15 highly conserved amino acids in the mature state, which participate in root development and other multiple physiological activities, such as symbiotic nitrogen fixation and nitrate uptake (Ohyama *et al.*, 2008; Imin *et al.*, 2013; Tabata *et al.*, 2014). These peptides are derived from non-functional pre-propeptides, processed by proteolytic cleavage and the addition of post-translational modifications (PTMs), and then transferred to the apoplast, stem xylem, or possibly the endoplasmic reticulum (ER), where they function as ligands with their corresponding receptors to trigger downstream responses (Okamoto *et al.*, 2016; Patel *et al.*, 2018; Zhu *et al.*, 2020). Genes from the *CEP* family are present across seed plants and have evolved via duplication and variation, which has led to the diversification of *CEP*-associated regulatory networks (Delay *et al.*, 2013a; Ogilvie *et al.*, 2014).

Previous studies on the *CEP* biology of non-legume species have usually used Arabidopsis as the research model and have mainly focused on regulation of the root architecture system and nitrogen demand signalling (Tabata *et al.*, 2014; Okamoto *et al.*, 2016; Taleski *et al.*, 2018). Fifteen *CEP* genes have been identified in the Arabidopsis genome (Roberts *et al.*, 2013), and the first identified gene was *CEP1*, which was identified using an *in silico* approach; this peptide repressed both primary and lateral root growth upon its overexpression or external application (Ohyama *et al.*, 2008). CEP receptors (CEPRs) have been subsequently characterized; these receptors perceive root-derived CEPs induced by nitrogen deficiency and trigger shoot to root nitrogen-demand reactions (Tabata *et al.*, 2014). Despite systematic regulation, CEP5 functions locally with CEPR1 in controlling lateral root initiation (Roberts *et al.*, 2016), and CEPs inhibit primary and lateral root growth in a CEPR1-dependent manner (Chapman *et al.*, 2019; Delay *et al.*, 2019).

An important property of legumes is their nodulation and nitrogen fixation capability, and the functions of CEPs in legumes warrant further investigation and discussion (Kereszt *et al.*, 2018). In the legume model plant *Medicago truncatula* Jemalong A17 Mt4.0 and Mt3.5v5 genome assemblies, 17 *CEP* genes have been identified by bioinformatic analysis (de Bang *et al.*, 2017); 13 of these *MtCEP* genes belong to group I and share similar CEP domains containing 15 amino acids, and the other four genes encode group II CEP domains that are unusual compared with group I CEPs, particularly in their N-terminal residues. *MtCEP1* encodes two CEP domains, modulates lateral root development, and enhances nodulation (Imin *et al.*, 2013). In addition, MtCEP7 has been demonstrated to enhance nodulation antagonistically to a CLE peptide systemic pathway that inhibits nodulation (Gautrat *et al.*, 2020; Laffont *et al.*, 2020). Using a modified isolation and characterization protocol, the presence of endogenous MtCEP1 peptides with distinct PTMs was validated, and the application of chemically synthesized peptides with different PTM patterns affected lateral root formation to varying degrees (Mohd-Radzman *et al.*, 2015). In addition, mass spectrum (MS) and bioinformatics approaches have been used to identify more PTM diversity among MtCEP1, 2, 5, and 8 *in vivo* (Patel *et al.*, 2018). COMPACT ROOT ARCHITECTURE 2 (CRA2) is referred to as the homologue of CEPR1 in *M. truncatula* and the candidate receptor for MtCEPs, but there is no biochemical proof that CEPs directly bind to CRA2 (Huault *et al.*, 2014; Mohd-Radzman *et al.*, 2015; Laffont *et al.*, 2019; Zhu *et al.*, 2020). A *cra2* mutant exhibits a particular phenotype in its belowground parts, and this phenotype is characterized by an increase in lateral roots that is not affected by the nitrogen or sucrose concentration and significantly inhibited nodulation under symbiotic conditions (Huault *et al.*, 2014). In addition, the *cra2* mutant is unresponsive to MtCEP1 treatment, which is suggestive of a ligand–receptor relationship (Mohd-Radzman *et al.*, 2016; Zhu *et al.*, 2020). However, few studies based on knockout mutants have identified the contribution of other *MtCEP* genes to root architecture and nodulation. The lack of *Mtcep* mutants has hindered further progress in this area.

The CRISPR/Cas 9 [clustered regularly interspaced short palindromic repeats (CRISPR)/CRISPR-associated protein 9] system is the most versatile genome editing tool developed over the last 30 years and has been widely used for single or multiple target(s) editing in soybean, wheat, cotton, maize, rice, etc. (Peng *et al.*, 2016; Zhang *et al.*, 2020). For the legume model plant *M. truncatula*, CRISPR/Cas9 techniques have been broadly applied for single-target mutagenesis (Meng *et al.*, 2017). Recently, a new multiplex genome editing system in tetraploid alfalfa (*Medicago sativa*) based on a polycistronic *tRNA*–*gRNA* approach was created by Wolabu *et al.* (2020), but still uses the *U6* promoter cloned from Arabidopsis and focuses on multiple sites of single gene editing. However, multigene editing remains difficult to perform, because few native *MtU6* promoters, but not *MtU6-1p*, have been confirmed to be valid for adequately driving small guide RNA (sgRNA) cassette expression in *M. truncatula*, which is necessary for multigene targeting in this species.

In this study, we identified 12 group I and four group II *MtCEP* genes in R108, and, herein we discuss the expression patterns of the group I *CEP* genes, and their functions during root development and nodulation. Quantitative reverse transcription–PCR (RT–qPCR) analysis showed that root-expressed *MtCEP1*, *2*, *4*, *5*, *6*, *7*, *8*, *9*, *12*, and *13* were up-regulated under nitrogen starvation conditions and that *MtCEP1*, *2*, *7*, *9*, and *12* were induced by rhizobial inoculation. In R108, the synthetic MtCEP1, 2, 4, 5, 6, 8, and 12 peptides reduced lateral root number and increased nodule number, whereas a *cra2* mutant was insensitive to these treatments. We modified the CRISPR/Cas9 toolbox (Xing *et al.*, 2014) for *M. truncatula* to generate the single-mutant lines *Mtcep1C* (C indicates that the mutant was created by CRISPR/Cas9), *Mtcep2C*, *Mtcep4C*, *Mtcep6C*, and *Mtcep12C*, and the double-mutant lines *Mtcep1*/*2C* and *Mtcep5*/*8C*, and these lines exhibited no visible changes in their phenotypes compared with that of the wild type (WT). We then knocked out *MtCEP1*, *2*, and *12* in both the WT and *Mtcep5*/*8C* backgrounds to obtain triple and quintuple *Mtcep* mutants, and found that these mutants exhibited higher numbers of lateral roots and fewer nodules, but did not recapitulate the *cra2* mutant phenotype. These results indicated that *MtCEP1*, *2*, and *12* are redundantly important for the coordination of lateral root formation and symbiotic nodulation, and the optimization of a CRISPR/Cas9 toolkit for multiplex genome editing could accelerate research on the functions of multigene families in *M. truncatula*.

## Materials and methods

### Plant materials and growth conditions

The *M. truncatula* R108 ecotype, which served as the WT in this study, was used as the genetic background for *Agrobacterium*-mediated transformation. The *cra2* (NF0903) mutant was obtained from the *Tnt1* insertion mutant library at the Noble Research Institute (Zhu *et al.*, 2020).

Seed sterilization was based on the procedure described in Duan *et al.* (2017). Briefly, the seeds were immersed in H_2_SO_4_ (98%, w/w) for 8 min, washed with ice-cold water three times, treated with 0.5% (v/v) NaClO for 12 min, and washed five times with sterile deionized water on a clean bench. After pre-treatment, the seeds were laid on 0.8% water agar medium and stored at 4 °C in darkness for 3 d. The seeds were then germinated at 25 °C under dark conditions for 12 h.

Plants were grown in a greenhouse at a temperature of 22 °C, a 16 h light/8 h dark photoperiod, a light intensity of 100–150 μmol s^–1^ m^–2^, and 60–70% humidity. In addition, plants were grown in a soil/perlite/vermiculite (1:2:5, v/v/v) mixture for seed production and in a perlite/vermiculite (2:5, v/v) mixture (imparts little resistance for root system penetration) saturated with nitrogen-depleted Fåhraeus liquid medium for nodulation. For root system architecture assessment, the matrix was removed with running water before the nodules and lateral roots were counted and the root length was measured and photographed.

### Bacterial materials and cultivation conditions for nodulation

*Sinorhizobium meliloti* strain 1021 (Sm1021) was used for symbiosis-associated experiments, and rhizobia cultured overnight were resuspended in sterile water (for inoculation on plates) or nitrogen-depleted Fåhraeus liquid medium (for inoculation in pots) at OD_600_=0.05 for inoculation. Seedlings grown aseptically on square plates for 7 d with Fåhraeus medium without nitrogen (13 cm×13 cm) were flooded with 20 ml of suspension for 1.5 h (each plate) (in contrast, 10 ml was used in Gonzalez-Rizzo *et al.*, 2006), and seedlings grown in pots in the greenhouse were subjected to injection with 10 ml (per plant) of suspension into the matrix (Gonzalez-Rizzo *et al.*, 2006).

### Effects of synthetic MtCEP peptides on root growth and nodulation

MtCEP peptides for external application were synthesized by GL Biochem (Shanghai, China) Ltd. MtCEP1, 2, 4, 5, 6, 8, and 12 were synthesized with hydroxylation at Pro4 and Pro11. When several conserved CEP domains existed in a precursor gene, the first of the CEP domains was selected for synthesis.

For the root growth analysis, each group of R108 and *cra2* mutants was grown on the same 1/2 Murashige and Skoog (MS, without sucrose, pH 5.8) solid medium (1.5% agar, w/w) supplemented with each synthetic MtCEP peptide at a concentration of 1 μM for 10 d, and the lateral roots were then counted for phenotyping.

For the nodulation analysis, the R108 and *cra2* mutants were first grown on nitrogen-depleted Fåhraeus solid medium covered with seed germination paper (Anchor Paper, www.anchorpaper.com) for 6 d and then transferred together with the germination paper to nitrogen-depleted Fåhraeus medium supplemented with 1 μM synthetic MtCEP peptide for an additional day. Inoculation with Sm1021 suspended in sterile water containing 1 μM synthetic MtCEP peptide (to maintain the concentration of CEP peptides in the germination paper) was performed at 7 d post-germination, and at 14 days post-inoculation (dpi) the nodules were counted for phenotype analysis.

### RT–qPCR assays of the expression of MtCEP genes in response to nitrogen starvation or rhizobial inoculation

The samples collected for RT–qPCR for the *MtCEP* expression analysis in R108 under nitrogen starvation conditions, or in response to rhizobial inoculation, were aseptically grown on plates. After germination, the seedlings were first laid on Fåhraeus solid medium with 10 mM NO_3_^−^ for 5 d, transferred to N-depleted Fåhraeus solid medium, and collected at 2 h, 12 h, 24 h, and 7 d after transition by dissecting the aboveground and belowground parts. For the nodulation analysis, the remaining seedlings were inoculated with Sm1021 after nitrogen starvation for 7 d; mixtures of roots and nodules from seven plants collected at 12 hours post-inoculation (hpi), 1 dpi, 3 dpi, 5 dpi, and 7 dpi acted as respective sample pools. For the expression of *MtCEP4*, *5*, *6*, *7*, *8*, *9*, *11*, and *13* in R108, *Mtcep1/2/12C*, and *Mtcep1/2/5/8/12C*, these seedlings were grown in liquid medium without nitrogen for 7 d (Zhu *et al.* 2020), and then the roots harvested from five plants were treated as one pool. Three independent pools of these tissues were used as three biological repetitions. TRIzol reagent (Invitrogen), trichloromethane, isopropanol, and ethyl alcohol (75%, v/v) were utilized for RNA extraction from the roots and shoots with overnight isopropanol precipitation (–20 °C), DNase (Promega, cat# M610A) was used to digest the DNA, and then the RNA was purified again with overnight isopropanol precipitation (–20 °C). Electrophoresis (1.2% agarose, v/v) was used to identify the RNA integrity, and a NanoDrop™ 2000 spectrophotometer was used to quantify the RNA concentration. Then, Moloney murine leukaemia virus (M-MLV) reverse transcriptase (Promega, cat# M1701) was used to synthesize first-strand cDNA from 4.0 μg of total RNA. RT–qPCR was carried out on a CFX-96 real-time system (Bio-Rad) with SYBR Premix Ex-Taq (TaKaRa, cat# RR420A). Gene expression was calculated using the 2^–ΔΔCT^ method. The expression data were normalized based on two reference genes *MtACTIN11* and *MtRBP1* (Laffont *et al.*, 2020). The primers used to test the expression levels of each *MtCEP* gene by RT–qPCR are listed in Supplementary Table S1.

### Constructs for GUS reporter lines and GUS staining

The promoter regions of *MtCEP1* (2.81 kb), *2* (2.25 kb), *4* (2.75 kb), *5* (2.87 kb), *6* (2.8 kb), *8* (2.91 kb), and *12* (2.77 kb) were cloned and ligated into pCAMBIA_1381 to drive the expression of the *GUS* (β-glucuronidase) gene. The primers for the constructs are listed in Supplementary Table S1. These constructs were transformed into R108 by *Agrobacterium tumefaciens* strain EHA105, which resulted in the generation of stable transgenic lines. Genomic DNA from the transgenic lines was extracted using the cetyltrimethylammonium bromide (CTAB) method (Springer, 2010), and the exogenous fragments from the plasmids inserted during the transformation were identified by PCR.

For GUS staining, the transgenic GUS reporter lines were grown in pots with nitrogen-depleted Fåhraeus medium for 7 d and then inoculated with Sm1021, and samples were collected at 0, 5, and 14 dpi (Fig. 2). For the GUS expression patterns at the early stage of rhizobial inoculation, the seedlings were grown in plates with Fåhraeus medium (without nitrogen) for 7 d and then inoculated with rhizobia, and GUS staining was carried out at 0, 1, and 2 dpi (Supplementary Fig. S4). Based on the method from Cervera (2005), the harvested material was immersed in staining buffer [50 mM phosphate buffer (pH 7.0), 1 mM 5-bromo-4-chloro-3-indolyl-β-d-glucuronic acid (X-Gluc), 5 mM potassium ferricyanide, and 5 mM potassium ferrocyanide], treated with a vacuum (>0.09 MPa) for 30 min, and stained at 37 °C for 6 h. The superfluous dye and chlorophyll were removed several times with 75% ethanol (6 h per wash), and the tissues were subsequently immersed in transparent liquid [4% Arabic gum (w/w), 60% trichloroacetic aldehyde (w/w), 3% glycerine (w/w)], and then imaged using an Olympus microscope (BX51) with an interference device.

### Optimization of the CRISPR/Cas9 genome editing protocol

We first cloned the *MtU6-1* promoter from A17 genomic DNA using the primer pair MtU6-1-F/R, and the *MtU6-3/5/6* promoter from R108 genomic DNA using the primer pairs MtU6-3-F/R, MtU6-5-F/R, and MtU6-6-F/R. For modification of the sgRNA cassette, we amplified the sgRNA scaffold and *AtU6-26* terminator from the original pCBC plasmid (Xing *et al.*, 2014) using the primer pairs pCBC-F and pCBC-MtU6-1/3/5/6-MR, conjoined this fragment with the *MtU6-1/3/5/6* promoter by overlapping PCR with the primer pairs pCBC-F and MtU6-1/3/5/6-R, and ligated the final long fragments into the pLB vector (TIANGEN, cat# VT206), which resulted in p1/3/5/6CBC.

We then amplified fragments of the *MtU6-3/6* promoter with the *Hin*dIII restriction enzyme cleavage site at the 5′ end and overlapping sequences of *SpR* at the 3′ end with the primer pairs HindIII-MtU6-3/6-F and MtU6-3/6-SpR-R, and fragments of the *SpR*+*sgRNA* scaffold+*AtU6-26* terminator from the original pHSE401 plasmid using the primer pairs MtU6-3/6-SpR-F and pHSE401-R. These two components were conjoined by overlapping PCR with the primer pairs HindIII-MtU6-3/6-F and pHSE401-R, and digested with *Hin*dIII. Additionally, pHSE401 was digested with *Hin*dIII, and the long fragment with the *Cas9* cassette and selectable marker *Hyg* cassette was retained. Fragments of the *MtU6-3*/*6* promoter+*SpR*+*sgRNA* scaffold+*AtU6-26* terminator were then inserted into digested pHSE401 to produce the binary vector pHSE3/6-401. All the primers used in this section are presented in Supplementary Table S1.

### Mutant generation using CRISPR/Cas9-based approaches

Based on Xing *et al.* (2014), sgRNA cassettes targeting *MtCEP* genes for the generation of single or double mutants were amplified from modified versions of p1/3/6CBC (~5 ng) by the primer pairs Target1-401-BsF/Target1-F0/Target2-1CBC-R0/Target2-BsR, Target1-401-BsF/Target1-F0/Target2-3CBC-R0/Target2-BsR, or Target1-401-BsF/Target1-F0/Target2-6CBC-R0/Target2-BsR, in which F0/R0 was diluted to 1 μM and BsF/BsR was diluted to 10 μM, and ligated into pHSE401 by the Golden Gate reaction [2 μl of pHSE401 (~200 ng μl^–1^), 8 μl of sgRNA cassette fragment (~100 ng μl^–1^), 1 μl of *Bsa*I (NEB, cat#R0535), 1 μl of T4 DNA ligase (NEB, cat#M0202T), 1.5 μl of 10× T4 DNA Ligase Buffer (NEB), and 1.5 μl of 10× BSA, 37 °C for 5 h, 50 °C for 5 min and 80 °C for 10 min]. The ligation products were transformed into *Escherichia coli* with resistance to kanamycin and the plasmids were extracted and confirmed by sequencing. The constructs were separately transformed into R108 with resistance to hygromycin by *A. tumefaciens* strain EHA105, and the regenerated transgenic lines were identified by PCR. The specific mutations were confirmed by amplifying the genomic fragments flanking the target sites, ligating them into intellectual vectors, and then sequencing randomly selected positive clones.

The construct targeting *MtCEP1/2/12* was based on the procedure described in Xing *et al.* (2014). Briefly, *sgRNA* cassettes were cloned from p1CBC and p6CBC with the addition of the corresponding target site by the primer pairs Target1-401-BsF/Target1-F0/1CBC-R0 and Target2-6CBC-BsF/Target2-F0/Target3-6CBC-R0/Target3-BsR, which resulted in p1CBC-MtCEP1-Target and p6CBC-MtCEP2*-*Target/MtCEP12*-*Target, respectively. The two fragments were simultaneously ligated into the binary vector pHSE401 by the Golden Gate reaction in a single round as described above. The construct was transformed into R108 and *Mtcep5/8* separately by EHA105 to generate *Mtcep1/2/12C* and *Mtcep1/2/5/8/12C*. The regenerated seedlings were tested by PCR using primer pairs for fragments of transferred T-DNA and genomic DNA flanking the target sites in *MtCEP1/2/12*, and the mutation types were identified by sequencing. The target sites selected for *STENOFOLIA* (*STF*) and *MtCEP* editing, and the corresponding primers are listed in Supplementary Table S2.

### Nodulation and root system architecture phenotype assays

Phenotype assays were conducted in a greenhouse. Seedlings of the mutants and the control group were planted in a matrix with nitrogen-depleted Fåhraeus liquid medium for 7 d and then inoculated with Sm1021. Various metrics of the root system architecture, including root lengths and lateral root number, were measured at 14 dpi, and the lateral root densities were calculated as the lateral root number divided by the root length, whereas the nodules were counted at 14 and 21 dpi.

## Results

### Different expression levels of *MtCEP* genes induced by nitrogen starvation or rhizobial inoculation

The *CEP* genes identified from the Jemalong A17 Mt4.0 and Mt3.5v5 genome assemblies can be divided into two groups depending on the conservation of amino acid sequences of their CEP domain: group I CEPs and group II CEPs (de Bang *et al.*, 2017). However, in the *M. truncatula* R108 genome and Jemalong A17 Mt5.0 genome assembly, 16 *MtCEP* genes, but not *MtCEP3*, were identified by comparison with the genomic database and by a transcriptomic assay, and *MtCEP10* encoded three identical CEP domains in R108. In addition, we discovered that these CEP precursor genes and their CEP domains were conserved between R108 and A17 (Supplementary Table S3). The top 12 *MtCEP* genes with high sequence similarity in their CEP domain belonged to group I, whereas the *MtCEP14–MtCEP17* genes, whose CEP domains contained unusual motifs compared with those of group I CEPs, were classified as group II (Supplementary Fig. S1A, B; Supplementary Table S3). Due to little evidence on the genetic functions of the group II CEPs and their association with group I functions, group II genes were excluded in this work. We conducted both *in vitro* and *in vivo* experiments to determine the functions of the group I *MtCEP* genes.

**Fig. 1.**
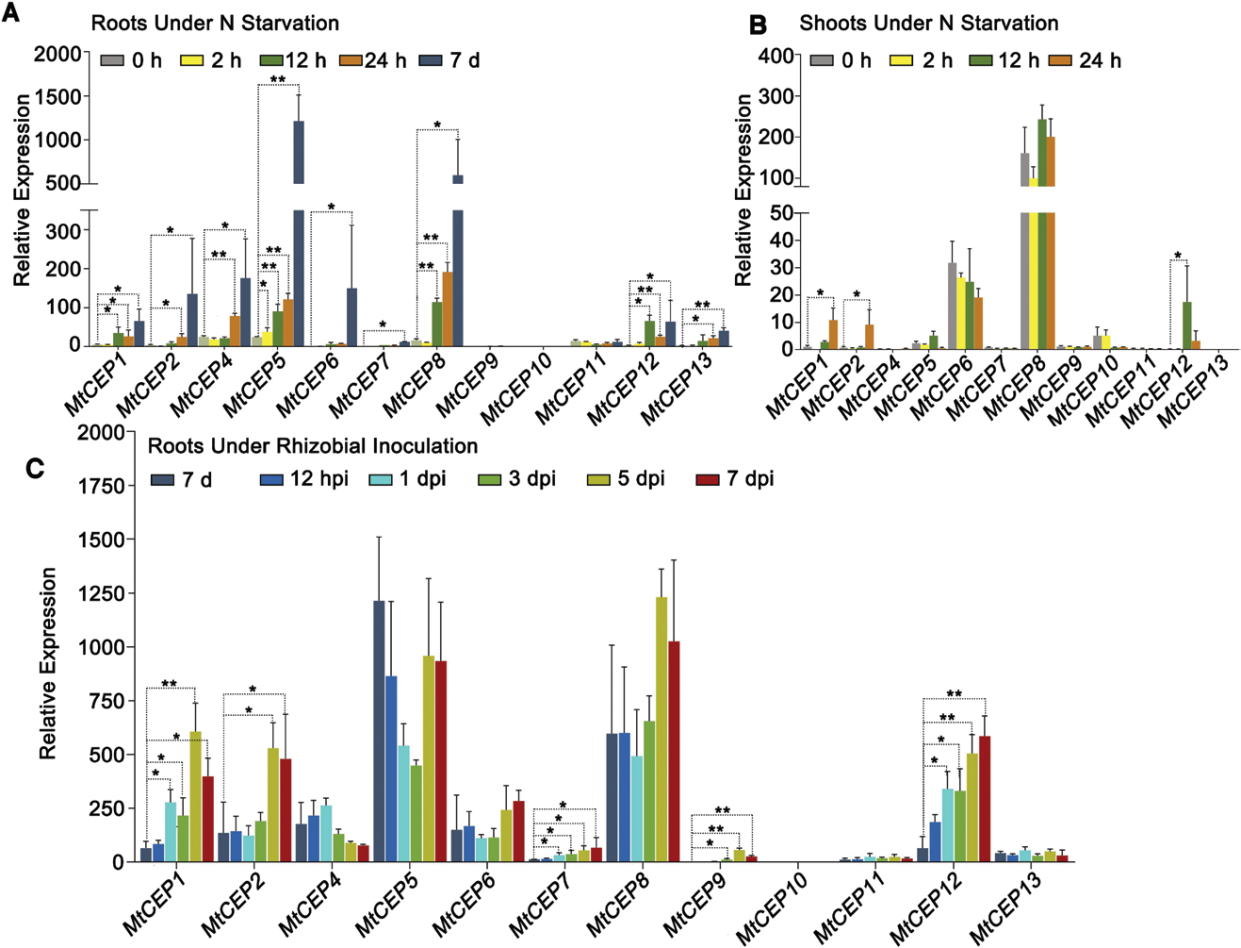
Relative expression levels of the group I *MtCEP* genes under nitrogen starvation conditions or in response to rhizobial inoculation. The germinated R108 seedlings were grown on Fåhraeus medium plates with 10 mM NO_3_^−^ for 5 d and then transferred to Fåhraeus medium without nitrogen, and the (A) roots and (B) shoots were harvested separately after 0, 2, 12, and 24 h, and 7 d. The remaining 12-day-old plants were inoculated with rhizobia; (C) the whole roots contained the primordium or nodules at 12 h post-inoculation (hpi) and 1, 3, 5, and 7 days post-inoculation (dpi). *MtACTIN11* and *MtRBP1* were used as the reference genes. The expression of *MtCEP1* in shoots at 0 h was normalized to 1, and the same scale on the *y*-axis was used in (A), (B), and (C). The data were derived from three independent pools of samples and are presented as the means± SDs (n=3). Significant differences were determined with a paired two-tailed Student’s *t*-test (*P<0.05, **P<0.01).

To investigate whether *MtCEP* genes were induced by low-nitrogen conditions, we laid germinated R108 seedlings on Fåhraeus medium plates with 10 mM NO_3_^−^ for 5 d and then transferred them to Fåhraeus medium without nitrogen. Roots and shoots were collected separately for RT–qPCR at 2, 12, and 24 h, and 7 d after starvation treatment. The relative expression levels of *MtCEP1*, *2*, *4*, *5*, *6*, *7*, *8*, *9*, *12*, and *13* in roots increased to varying degrees, and, among these, *MtCEP5* and *MtCEP8* exhibited the strongest expression, and *MtCEP7* and *MtCEP9* showed very low expression levels (Fig. 1A; Supplementary Fig. S2A). Although mild elevations in the relative expression levels of *MtCEP1*, *2*, and *12* were found in shoots, these *CEP* genes showed poor expression in shoots, with the exception of *MtCEP8* (Fig. 1B).

To test whether *MtCEP* genes were induced by rhizobial inoculation, R108 seedlings subjected to nitrogen starvation for 7 d were inoculated with Sm1021 on plates. Root samples were collected at 12 hpi, and 1, 3, 5, and 7 dpi for RT–qPCR. After inoculation, the relative expression levels of *MtCEP1*, *7*, 9, and *12* were up-regulated at an early stage (1 and 3 dpi), and that of *MtCEP2* was up-regulated at a later stage (5 and 7 dpi). However, the expression levels of *MtCEP7* and *MtCEP9* were much lower than those of *MtCEP1*, *2*, and *12* (Fig. 1C; Supplementary Fig. S2B). Additionally, our previous RNA-seq data on roots and shoots of R108 inoculated with rhizobia for 5 d (Zhu *et al.*, 2020) revealed that *MtCEP1*, *2*, *4*, *5*, *6*, *8*, and *12* presented higher expression in roots among the 12 *MtCEP* genes (Supplementary Fig. S3). We then focused on these seven *MtCEP* genes that were induced by nitrogen starvation or rhizobial inoculation.

### Spatial expression patterns of *MtCEP* genes

**Fig. 2.**
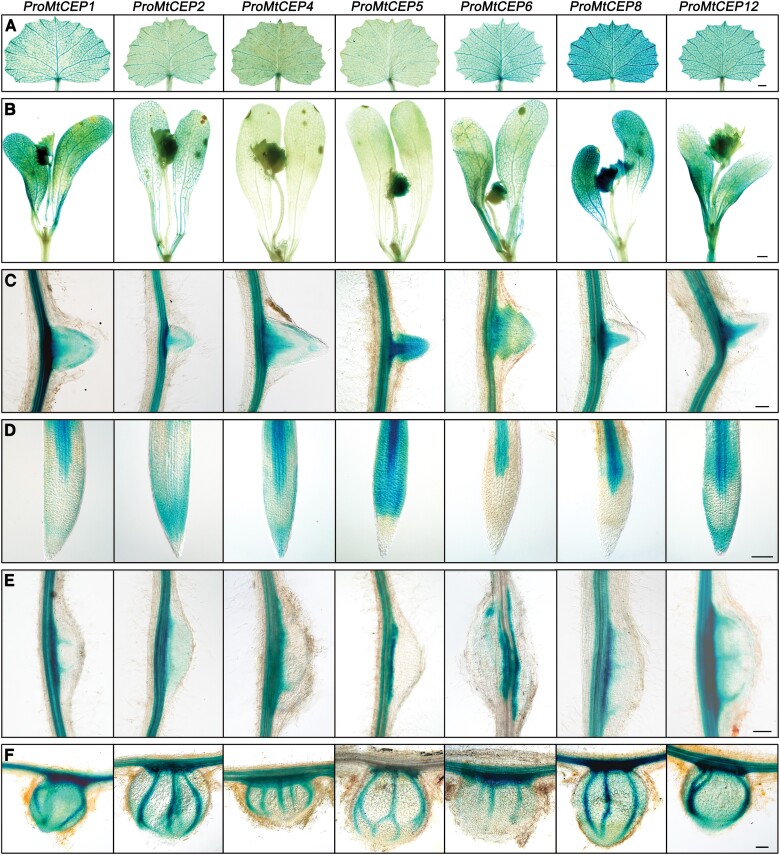
Spatial expression patterns of *MtCEP* genes. The 2.2–3 kb promoters of *MtCEP1*, *MtCEP2*, *MtCEP4*, *MtCEP5*, *MtCEP6*, *MtCEP8*, and *MtCEP12* were cloned into *pCAMBIA*_1381 to drive the expression of *GUS*, and the fused vectors were then stably transferred to R108. The stable transgenic plants *ProMtCEP1*:*GUS*, *ProMtCEP2*:*GUS*, *ProMtCEP4*:*GUS*, *ProMtCEP5*:*GUS*, *ProMtCEP6*:*GUS*, *ProMtCEP8*:*GUS*, and *ProMtCEP12*:*GUS* were germinated and grown in a soil/vermiculite mixture with Fåhraeus medium without nitrogen and inoculated with rhizobia 7 d later. The GUS staining images of the first single leaf (A), first compound leaves and two cotyledons (B), emerging lateral roots (C), primary root tips (D), and nodule primordium (E) at 5 dpi and the nodules (F) at 14 dpi are shown. The scale bar represents 1 mm in (A) and (B), and 200 µm in (C–F).

To further investigate the spatial expression patterns of the candidate *MtCEP* genes, we cloned the 2.2–3 kb promoters of *MtCEP1*, *2*, *4*, *5*, *6*, *8*, and *12* and fused them with the *GUS A* coding region in the backbone of pCAMBIA_1381. We generated stable transgenic GUS reporter lines in the R108 background, and these were termed *ProMtCEP1*:*GUS*, *ProMtCEP2*:*GUS*, *ProMtCEP4*:*GUS*, *ProMtCEP5*:*GUS*, *ProMtCEP6*:*GUS*, *ProMtCEP8*:*GUS*, and *ProMtCEP12*:*GUS*. Seedlings of each line were grown in a mixture of vermiculite and perlite filled with liquid Fåhraeus medium without nitrogen for 7 d and then inoculated with rhizobia. GUS staining showed that these selected *MtCEP* genes exhibited diverse expression patterns. Although all of the genes were expressed in roots, differences were found between each of these *MtCEP* genes. For example, all seven *MtCEP* genes showed obvious staining in the stele of the primary root tip, whereas the staining signals of *ProMtCEP2*:*GUS*, *ProMtCEP4*:*GUS*, and *ProMtCEP12*:*GUS* were additionally reflected in the root cap (Fig. 2D). All seven *MtCEP* genes were expressed in the stele of the lateral root primordium, but *MtCEP1*, *2*, *4*, *5*, and *6* also showed diffuse staining in the periphery (Fig. 2C). During nodulation, *MtCEP1*, *2*, *4*, *5*, *6*, *8*, and *12* were first expressed in the vascular tissue, located in the base of the nodule primordium, and then expressed in vascular bundles surrounding the established nodule (Fig. 2E, F). In addition, unlike *MtCEP7* expressed in the root epidermis and infected cells during the early stage of rhizobial inoculation (Laffont *et al.*, 2020), all seven *MtCEP* genes were expressed in the vascular tissues of roots and showed no signals in the epidermis at 1 and 2 dpi (Supplementary Fig. S4). Staining in shoots was also observed. These results showed that *MtCEP1*, *2*, *5*, *6*, *8*, and *12* presented staining signals in cotyledons, whereas in true leaves, *MtCEP1*, *6*, *8*, and *12* exhibited clearly detectable GUS signalling in the first single leaf, and all these genes were expressed in the first compound leaves (Fig. 2A, B). In summary, these selected *MtCEP* genes presented redundant spatial expression patterns.

### External application of chemically synthesized MtCEP peptides led to fewer lateral roots and more nodules

To test the effects of different MtCEP treatments on nodulation or lateral root number, we chemically synthesized peptides of the seven candidate *MtCEP* genes that exhibited up-regulated expression under nitrogen starvation conditions or in response to rhizobial inoculation. According to MS analysis of CEP peptides by Tabata *et al.* (2014) and Patel *et al.* (2018), CEP peptides have variant derivatives with different hydroxylation patterns at proline residues 4, 7, and 11. For example, the MtCEP1a CEP domain with hydroxylation at Pro4 and Pro11 was the most biologically active variant of MtCEP1 (Patel *et al.*, 2018). Based on this information, MtCEP1, 2, 4, 5, 6, 8, and 12 were synthesized with hydroxylation at Pro4 and Pro11.

**Fig. 3.**
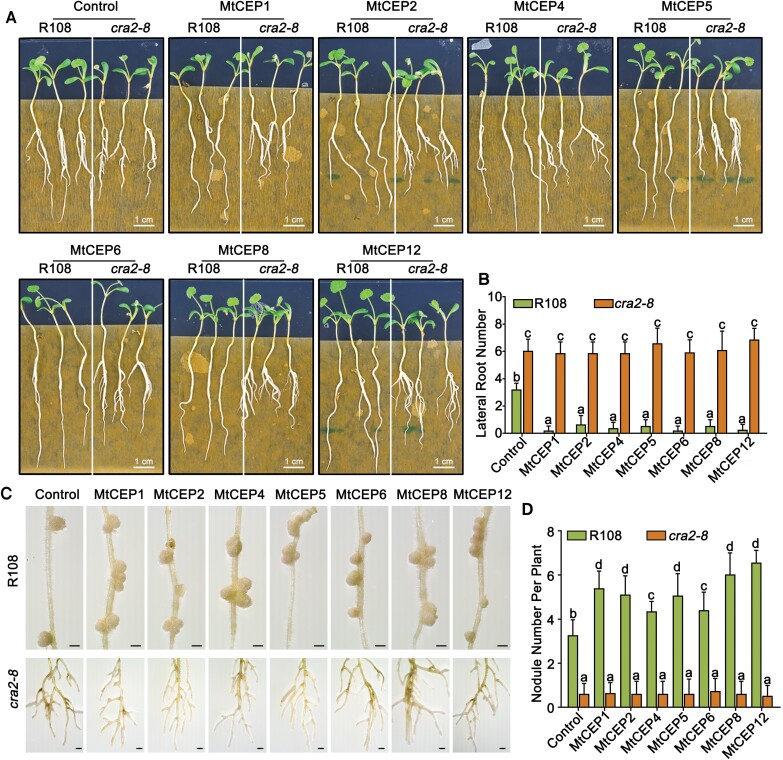
The external application of chemically synthesized MtCEPs led to fewer lateral roots and promoted nodulation. (A and B) Effects of chemically synthesized MtCEPs on lateral root development. Germinated R108 and *cra2-8* plants were grown in plates containing solidified 1/2 strength MS medium (without sucrose) with or without (control) 1 μM CEPs for 10 d. Representative images of root growth (A) and quantification of the lateral root number (B). Similar results were obtained in three independent experiments. The data are presented as the means ±SDs from one representative experiment, and significant differences were determined by ANOVA with Duncan’s post-hoc test, as indicated by letters (*P*<0.05, *n*=18). (C and D) Effect of chemically synthesized MtCEPs on symbiotic nodulation. The germinated R108 and *cra2-8* plants were grown in plates with Fåhraeus medium without nitrogen for 6 d and then transferred to new plates with Fåhraeus medium without nitrogen containing 0 (control) or 1 μM CEPs. One day later, these plants were inoculated with rhizobia. Representative images of R108 and *cra2-8* plants (C) and quantification of the nodule number at 14 dpi (D). Similar results were obtained in three independent experiments. The data are presented as the means ±SDs from one representative experiment, and significant differences were determined by ANOVA with Duncan’s post-hoc test, as indicated by letters (*P*<0.05, *n*=18). The scale bar in (C) represents 1 mm.

To determine the effects of the MtCEPs on lateral root development, the R108 WT and *cra2* mutant lines were grown on the same 1/2 MS medium supplemented with or without each MtCEP at a concentration of 1 μM, and the lateral root number was counted 10 d after germination. All the groups of R108 treated with MtCEPs had fewer lateral roots than the control, but no significant difference was found among the *cra2* mutant groups. This result suggested that these artificial MtCEPs decreased lateral root number (Fig. 3A, B).

For the nodulation assay, R108 and *cra2* mutant lines were first planted on Fåhraeus medium without nitrogen or CEP addition, and 6 d later, the plants were transferred to nitrogen-free Fåhraeus medium containing 1 μM CEP. One day later, all the groups were inoculated with Sm1021, and the nodules were counted 2 weeks after inoculation. R108 treated with MtCEP1, 2, 4, 5, 6, 8, and 12 had more nodules than the control, and all *cra2* mutants subjected to CEP addition or treated with only water had far fewer nodules than WT plants (Fig. 3C, D). This result suggested that these seven MtCEPs contribute to the nodulation process.

### Optimization of the CRISPR/Cas9 genome editing protocol

Although CRISPR/Cas9 technology has been utilized for targeted mutagenesis in *M. truncatula*, the present system is not sufficient for multigene editing (Meng *et al.*, 2017; Wolabu *et al.*, 2020). We first tested the original CRISPR/Cas9 toolkit for Arabidopsis (Xing *et al.*, 2014) with the target gene *STF* in *M. truncatula* R108, as the knockout mutation exhibits apparently visible phenotype changes in young seedlings (Tadege *et al.*, 2011). A total of 271 transgenic seedlings were generated by *Agrobacterium*-mediated transformation in the R108 genetic background, and ~8% (22/271) of the seedlings showed spindly leaves. Sequencing analysis showed that all the mutations (deletions or insertions) (22/22) occurred at the first target site driven by *AtU6-26p*, but none (0/22) of the mutations occurred at the second target site driven by *AtU6-29p*, which indicated the existence of some adapted modifications of this toolkit for a new species (Supplementary Fig. S5).

**Fig. 4.**
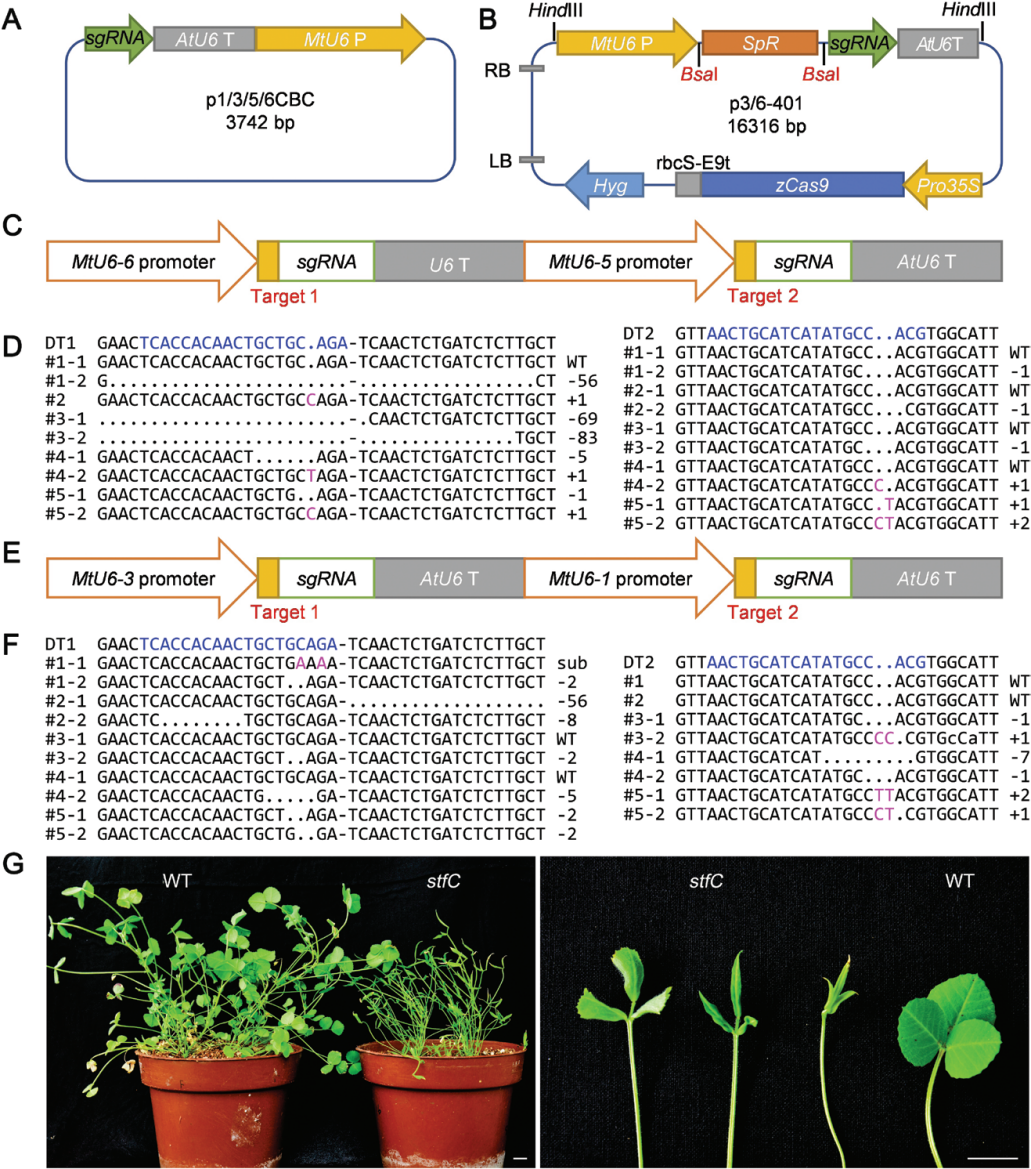
Multiple-target mutagenesis of *M. truncatula* using the modified CRISPR/Cas9 toolbox. (A) Structure model of p1/3/5/6CBC based on the pLB backbone. The original *gRNA* cassette was cloned from pCBC, and *MtU6-1*/*3*/*5*/*6p* replaced the original *AtU6-29p*. The whole fragment was ligated into the commercial blunt-ended vector pLB. (B) Structure model of optimized p3/6-401 based on the pHSE401 backbone. *MtU6-3p* and *MtU6-6p* replaced the original *AtU6-26p* without changing the basic vector backbone. *Zea mays* codon-modified *zCas9* was driven by the 2×35S promoter. The hygromycin resistance gene was used as a selectable marker for plant transformation, and the kanamycin resistance gene was used for plasmid propagation. (C–F) Illustration of the modified sgRNA cassettes in p6401-5CBC-*STF* (C) and p3401-1CBC-*STF* (E), and mutagenesis at target 1 and target 2 of *STF* generated using the p6401-5CBC-*STF* (D) and p3401-1CBC-*STF* (F) CRISPR/Cas9 systems. The blue letters represent the target sites, and the magenta letters represent the inserted or mismatched sites. Sub, substitution. (G) Phenotype of *stf* mutants transferred to soil and grown for 1 month. The scale bar represents 1 cm. The ‘–’ in (D) and (F) represents a sequence of 17 bp. P, promoter; T, terminator.

We therefore cloned the *MtU6-1* promoter from the A17 ecotype, which is reportedly efficient, and another three *MtU6* promoters from the R108 ecotype (Supplementary Table S4), designated *MtU6-3p*, *MtU6-5p*, and *MtU6-6p*. The original *AtU6-26p* in pHSE401 was replaced by *MtU6-3p* and *MtU6-6p* to yield p3401 and p6401 (Fig. 4A), respectively, whereas *AtU6-29p* in pCBC was substituted with *MtU6-1p*, *MtU6-3p*, *MtU6-5p*, and *MtU6-6p* to obtain p1CBC, p3CBC, p5CBC, and p6CBC, respectively (Fig. 4B). The p3401-1/5/6CBC and p6401-1/3/5CBC pairs can simultaneously target at most four sites in the genome of *M. truncatula* (Fig. 4A, B).

The modified system for single- or double-site editing was similarly tested by targeting *STF* in *M. truncatula*. The new target sites were fused into constructs to yield p3401-1CBC-*STF* and p6401-5CBC-*STF*. Transgenic lines were generated by stable transformation, and 40% (24/60) of the seedlings transformed with p3401-1CBC-*STF* and 43% (19/44) of the seedlings transformed with p6401-5CBC-*STF* displayed spindle-like leaves as early as the regeneration stage. To determine the exact mutations of each target, we randomly selected five transgenic lines with an *stf*-like phenotype from each transformation event, and a sequence analysis showed that mutations occurred at both target sites, which confirmed the efficiency of each native *MtU6* promoter. The analysis of the transgenic lines regenerated by the transformation of p6401-5CBC-*STF* revealed that the first target sites driven by the *MtU6-6* promoter were all mutated; among these, #2–5 were biallelic or homozygous mutations at target 1, whereas #1 was heterozygous with a WT allele (Fig. 4C, D). Although the second target sites driven by the *MtU6-5* promoter were all also edited, four out of five plants retained a WT allele (Fig. 4D). The analysis of the transgenic lines derived from the transformation event of p3401-1CBC-*STF* showed that #1–4 at target 1 driven by the *MtU6-3* promoter were heterozygous and that #5 at target 1 was biallelic. The second targets driven by the *MtU6-1* promoter, which has been previously reported, were edited only in three transgenic lines (Fig. 4E, F). These results suggested that the four *MtU6* promoters with validated efficiency in *STF* editing might be used for multitarget editing.

### Selected single or double mutations of *MtCEP* genes had no significant influence on root development or nodulation

**Fig. 5.**
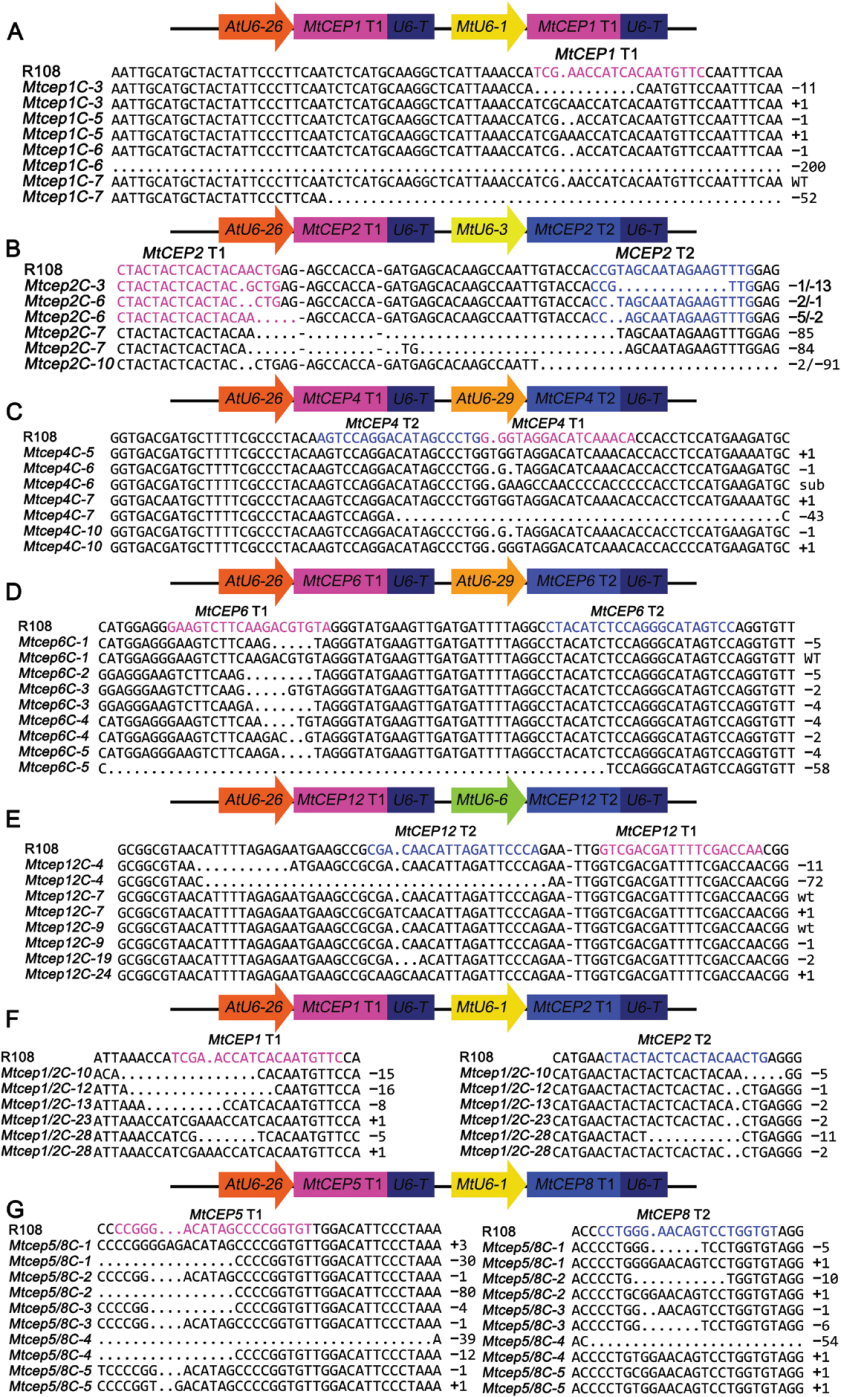
Generation of single or double mutants of *MtCEP* genes. Construction of *sgRNA* cassettes containing one or two target sites and the corresponding promoter for the single or double mutation and representative mutant sequences of the target site for single mutations of *MtCEP1* (A), *MtCEP2* (B), *MtCEP4* (C), *MtCEP6* (D), and *MtCEP12* (E), and double mutations of *MtCEP1*/*2* (F) and *MtCEP5*/*8* (G). The magenta letters represent target 1, and the blue letters represent target 2. T1 and T2 represent target 1 and target 2, respectively.

Due to the difficulty in discerning functional differences among MtCEP peptides based on largely overlapping spatial expression patterns, and on the results of the treatment with chemically synthesized MtCEP peptides, it is necessary to construct stable mutant lines for further exploration. Based on the optimized CRISPR/Cas9 system and *Agrobacterium*-mediated transformation in R108, several single-mutant lines *Mtcep1C*, *Mtcep2C*, *Mtcep4C*, *Mtcep6C*, and *Mtcep12C* were generated, and two double-mutant combinations *Mtcep1*/*2C* (initially, the triple mutant of *MtCEP1*, *2*, and *12* induced by both rhizobial inoculation and nitrogen starvation was planned, but the vector construction failed at the initial stage; therefore, *Mtcep1*/*2C* was created) and *Mtcep5/8C* (the double mutant was used instead of a single mutant for the preliminary functional analysis of *MtCEP5* and *8*, which have similar expression patterns) were selected for generation. Sequencing of four or five transgenic seedlings for each construct showed that the frequency of the homozygous and biallelic mutants for all the targets (except target 1 of *MtCEP12* driven by *AtU6-26p* and target 2 of *MtCEP4* or *MtCEP6* driven by *AtU6-29p*) was >60% (Fig. 5).

We then attempted to determine whether any of these single or double mutants had an effect on lateral root development or nodulation. The mutants were planted in a greenhouse for 7 d of nitrogen starvation. The seedlings were then inoculated with Sm1021 and grown for an additional 2 weeks. Several metrics relevant to common root development and nodulation processes were measured. No significant differences in the root length, lateral root number, lateral root density, or nodule number per plant were found between any of these single or double mutants and WT plants, and no other abnormal developmental phenotypes were observed (Supplementary Fig. S6, S7). Because overexpression of *MtCEP1* or chemical treatments inhibited lateral roots and promoted nodulation (Imin *et al.*, 2013), and based on the extensive overlapping patterns of *MtCEP* genes, all these results suggested that the functions of *MtCEP* genes in root development and nodulation might be highly redundant.

### Triple and quintuple mutations of *MtCEP* genes led to more lateral roots and fewer nodules

**Fig. 6.**
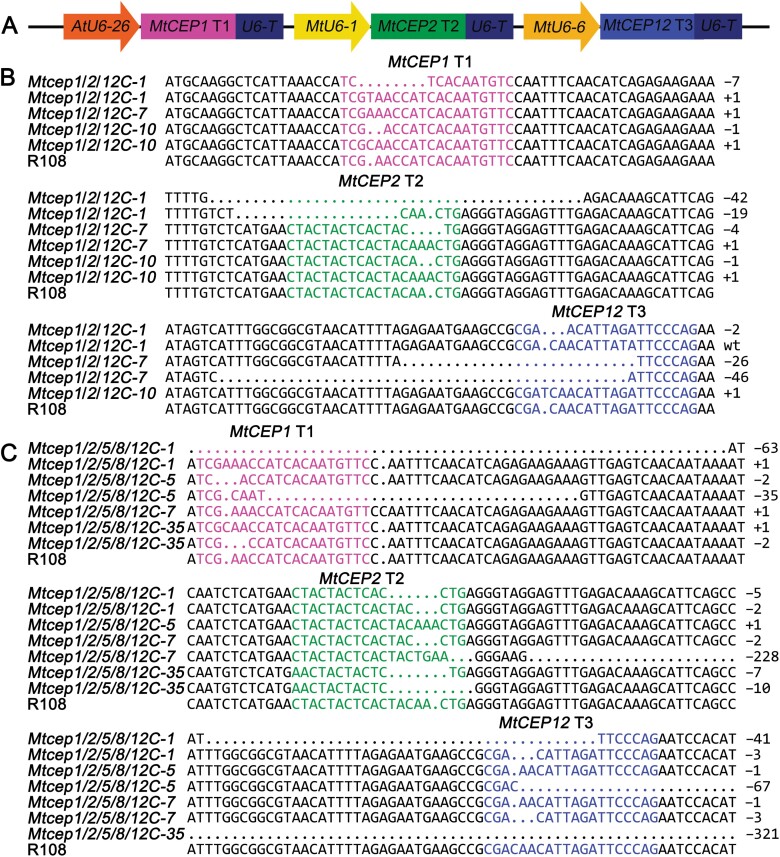
Generation of triple and quintuple mutants of *MtCEP* genes. (A) Construction of sgRNA cassettes containing triple target sites for *MtCEP1*, *MtCEP2*, and *MtCEP12*. For the triple mutant *Mtcep1*/*2*/*12C*, the CRISPR/Cas9 platform was transformed into R108, and for the quintuple mutant *Mtcep1*/*2*/*5*/*8*/*12C*, this vector was transformed into *Mtcep5*/*8C*. Representative mutant sequences of the target sites for the triple mutant *Mtcep1*/*2*/*12C* (B) and the quintuple mutant *Mtcep1*/*2*/*5*/*8*/*12C* (C). The magenta letters represent target 1, the cyan letters represent target 2, and the blue letters represent target 3. T1, T2, and T3 represent target 1, target 2, and target 3, respectively.

To determine the degree of redundancy among the members of the *MtCEP* gene family, we subsequently utilized CRISPR/Cas9 technology and simultaneously targeted *MtCEP1*, *MtCEP2*, and *MtCEP12*. We generated triple and quintuple mutants through *Agrobacterium*-mediated transformation in the WT and the already available *Mtcep5/8C* double mutant genetic backgrounds, respectively (Fig. 6A, B). The specific mutations in each target were confirmed by PCR and sequencing, and the results showed that the frequencies of homozygous and biallelic mutations in *MtCEP1*, *MtCEP2*, and *MtCEP12* were 52% (12/23), 59% (13/22), and 48% (10/21), respectively (Fig. 6C, D). Additionally, RT–qPCR indicated that the expression levels of *MtCEP4*, *5*, *6*, *7*, *8*, *9*, *10*, and *11* were not significantly different among R108, *Mtcep1*/*2*/*12C*, and *Mtcep1*/*2*/*5*/*8*/*12C* (Supplementary Fig. S8). These results suggested that mutations of the five *MtCEP* genes showed no feedback effects on the expression of the other *MtCEP* genes.

**Fig. 7.**
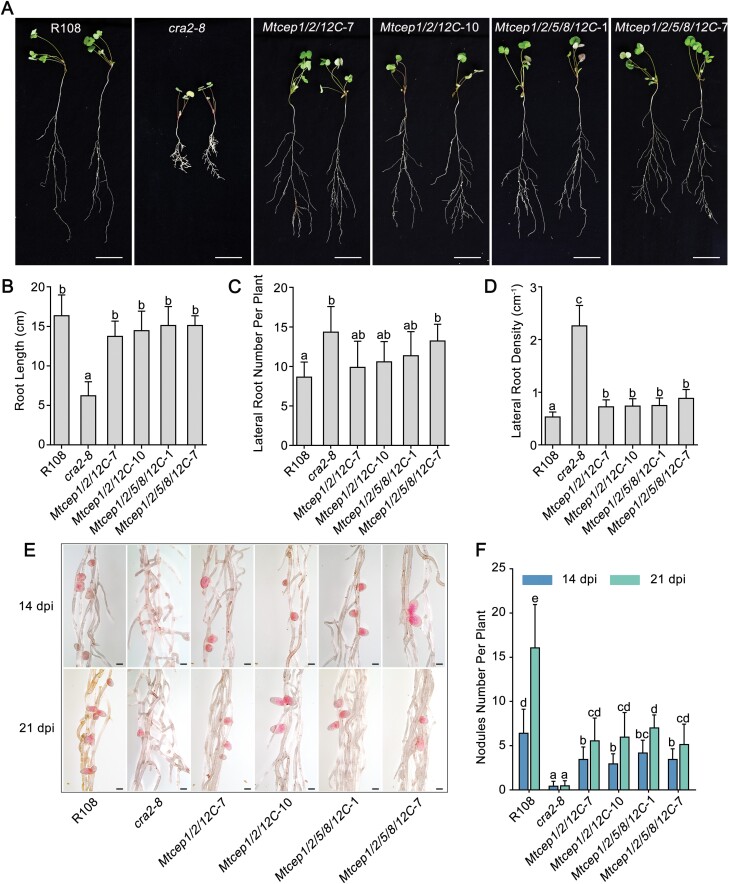
*MtCEP1*, *2*, and *12* play important roles in regulating lateral root development and symbiotic root nodulation. Root growth and nodulation phenotypes of R108, *Mtcep1*/*2*/*12C*, and *Mtcep1*/*2*/*5*/*8*/*12C* inoculated with Sm1021 after 1 week of growth in a perlite/vermiculite mixture with nitrogen-free Fåhraeus medium. Representative images of whole plants at 14 dpi (A) and quantification of the root length (B), lateral root number (C), and lateral root density (D). The scale bar represents 2.5 cm. Representative images of the nodulation phenotype at 14 and 21 dpi (E) and quantification of the nodule number at 14 and 21 dpi (F). Similar results were obtained in three independent experiments; the data are presented as the means ±SDs from one representative experiment, and significant differences were determined by ANOVA with a post-hoc LSD test, as indicated by letters (*P*<0.05, *n*>15). The scale bar represents 1 mm.

For the root and nodule phenotypic analysis, *Mtcep1/2/12C*, *Mtcep1/2/5/8/12C*, R108, and the *cra2* mutant were subjected to nitrogen starvation for 7 d and then to rhizobial inoculation in a greenhouse. At 14 dpi, we measured the root length of each line and counted the lateral root number per plant. Although the root length and lateral root number of the triple and quintuple mutants were slightly but not significantly changed compared with those of the WT plants, the lateral root densities of the multigene mutants were higher than those of R108 (Fig. 7A–D), which suggested that lateral root densities were promoted in *Mtcep1/2/12C* and *Mtcep1/2/5/8/12C*. With respect to nodulation, the nodule number counted at 14 and 21 dpi in the triple and quintuple *Mtcep* mutants was lower than that in R108, but the lateral root and nodulation phenotype of *Mtcep1/2/12C* and *Mtcep1/2/5/8/12C* did not reach the abnormal degree of the *cra2* mutant phenotype (Fig. 7E, F). In addition, the nodule numbers of *Mtcep1/2/12C* and *Mtcep1/2/5/8/12C* were not significantly different from each other. These results suggested that at least *MtCEP1*, *MtCEP2*, and *MtCEP12* act redundantly in controlling lateral root and nodule number, whereas *MtCEP5* and *MtCEP8* have no major role.

## Discussion

The CEP peptide and its putative receptor CRA2 are thought to be the central regulatory components involved in balancing root architecture and nodulation under nitrogen-limited conditions (Imin *et al.*, 2013; Mohd-Radzman *et al.*, 2016; Patel *et al.*, 2018; Laffont *et al.*, 2020; Zhu *et al.*, 2020). Although an external addition assay of MtCEPs was conducted by Imin *et al.* (2013) (MtCEP1), Patel *et al.* (2018) (MtCEP1, 2, 5, and 8), and Laffont *et al.* (2020) (MtCEP7), we conducted an external application experiment using seven group I MtCEPs that were chemically synthesized with presumed PTMs. The functional analysis suggested that these synthetic peptides had biological activities in lateral root inhibition and promoting nodulation (Fig. 3). However, these results could not distinguish the specific functions of these MtCEPs because of their highly conserved amino acid sequences. Furthermore, the PTM patterns of synthetic peptides may not represent the native circumstances. Recently, *in vivo* data for *Medicago* or soybean revealed another hydroxylated proline combination (Mohd-Radzman *et al.*, 2015; Okamoto *et al.*, 2015; Patel *et al.*, 2018). Additionally, Patel *et al.* (2018) showed that MtCEP2 hydroxylated at Pro4 and Pro11 did not exert a positive effect on nodulation. However, in contrast, we observed a promoting effect of MtCEP2–MtCEP4, 11-hydroxylated peptides, and this difference may be explained by the different *Medicago* ecotypes used, the treatment pattern (e.g. duration of CEP treatment/pre-treatment), or other experimental details (e.g. inoculation method). Previous studies have revealed that *cra2* could not respond to MtCEP1 and MtCEP7 peptides (Mohd-Radzman *et al.*, 2016; Laffont *et al.*, 2020). Consistent with that, the *cra2* mutant could not respond to any of the tested MtCEPs in our study (Fig. 3), and this finding suggests a CEP–CRA2 ligand–receptor relationship.

A few studies have performed high-throughput gene expression profiling at different stages of nodulation and in response to the elimination or limitation of macronutrient elements (Larrainzar *et al.*, 2015; de Bang *et al.*, 2017). In this study, the *MtCEP* gene expression patterns were validated in a low-throughput manner by RT–qPCR and the GUS reporter system (Figs 1, 2). However, there were some discrepancies between the GUS staining and RT–qPCR results. For example, RT–qPCR analysis showed that the relative expression level of *MtCEP6* was obviously higher than that of *MtCEP1* in shoots, regardless of the nitrogen conditions, but GUS staining revealed that the patterns in the aboveground parts of the GUS reporter lines driven by *MtCEP6* and *MtCEP1* were similar (Figs 1B, 2). The different growth conditions might have led to the differences. The samples collected for RT–qPCR were grown on plates with 10 mM NO_3_^–^ for 5 d and then transferred to medium without nitrogen, whereas the transgenic lines for GUS staining were grown in a matrix without nitrogen in a greenhouse for 7 d, so that excess nitrate stored in shoots and cotyledons would provide a nitrogen source for plant growth, which might delay the response to low-nitrogen conditions in shoots and roots in the RT–qPCR assay. Additionally, the cloned promoters might not represent *in vivo* expression adequately. Furthermore, the clearly detectable GUS signalling of *MtCEP1*, *6*, and *12* suggested these *MtCEP* genes may play some roles in shoot development regulation; as in Arabidopsis, *AtCEP5* expression in the shoot apical meristem and cotyledon is involved in aboveground growth regulation (Roberts *et al.*, 2013). Imin *et al.* (2013) performed a detailed analysis of the expression pattern of *MtCEP1* in transgenic roots expressing *ProMtCEP1*:*GUS* generated by hairy root transformation and found that staining was diminished in developing nodules and even absent in mature nodules (14 dpi). However, these phenomena were not observed in this study, in which stably inherited transgenic lines (14 dpi) were used as the experimental materials (Fig. 2F). The different genotypes used and the 3–4 week transformation process before rhizobial inoculation of hairy roots may cause the different expression patterns.

*Tnt1* insertion mutants have been utilized extensively for the study of gene function in *M. truncatula*; however, when multiple mutants are required for genetic functional analyses, especially when genes are clustered in the same genomic region, as several CEP genes are (e.g. *MtCEP1*, *2*, and *8* are clustered on chromosome 8), the CRISPR strategy is faster and more convenient than crossing *Tnt1* mutants. However, the existing CRISPR/Cas9 technology was utilized only for single-target mutagenesis in *M. truncatula* (Meng *et al.*, 2017). Based on the CRISPR/Cas9 toolkit for Arabidopsis, we cloned the *MtU6-1*, *3*, *5,* and *6* promoters from *M. truncatula* and optimized the original CRISPR/Cas9 system with these four promoters (Fig. 4A, B). The modified CRISPR/Cas9 system was proven to be very efficient in *STF* editing and in the generation of single or double *MtCEP* mutants (Figs 5, 6). Moreover, the frequency of homozygous and biallelic mutations for each target was >48% in *MtCEP1/2/12* triple-target editing (Supplementary Table S5).

To date, to the best of our knowledge, a null-allele *Mtcep* mutant has not been reported, which leads to a gap in knowledge regarding its function. Although RNAi-mediated or miRNA-induced gene silencing was applied to generate knockdown mutants targeting *MtCEP1*/*2*/*5*/*11*, the efficiency and specificity of both methods seemed limited (Imin *et al.*, 2013). The transgenic roots with declining expression levels of *MtCEP1* and *MtCEP2* had more lateral roots but an equivalent number of nodules (Imin *et al.*, 2013). However, in this study, the *Mtcep1*/*2C* double mutants generated using CRISPR/Cas9 technology showed no obvious difference in the phenotypes regarding either lateral root number or nodulation (Supplementary Figs S6, S7). The differences between the two studies are possibly due to the RNAi construct targeting other *CEP* genes (e.g. *CEP12*) that were not analysed by RT–qPCR. This finding again highlighted the necessity of specific knockout mutants for gene function studies.

Although these CEP genes targeted by CRISPR/Cas9 were selected based on their expression patterns and intensity, this combination of targets might not be the optimal solution because the *cra2* mutant presented a more remarkably extreme phenotype than the triple or quintuple *Mtcep* mutants (Fig. 7). A recent study indicated that *MtCEP7* could be induced by rhizobia, and the silencing of *MtCEP7* by RNAi or by an amiRNA significantly reduced the nodule number (Laffont *et al.*, 2020). However, *MtCEP7* and *MtCEP9*, which could actively respond to rhizobial inoculation, as demonstrated by our RT–qPCR data (Fig. 1), were excluded in the combinations of targets for CRISPR/Cas9 due to their low expression abundance. In addition, only the *Mtcep1*/*2C* double mutant was assayed, so additional combinations of mutants were needed to verify whether *MtCEP1*/*12* or *MtCEP2*/*12* might play more important roles, and whether any other genes played an additional role to that of *MtCEP1*/*2*/*12* in the nodulation process. Together, these results provide a promising method for creating multiple mutants of *MtCEP* genes, and these mutants expand our knowledge of CEP biology and improve our understanding of the complexity underlying the regulation of root system architecture in legumes.

## Supplementary data

The following supplementary data are available at *JXB* online.

Fig. S1. Representative scheme of the precursor protein and sequence alignment of CEP domains of the group I CEPs in R108.

Fig. S2. Fold change in the relative expression of group I *Mt*C*EP* genes under nitrogen starvation conditions or in response to rhizobial inoculation.

Fig. S3. Expression of *MtCEP* genes in roots and shoots of R108 determined by RNA-seq.

Fig. S4. Spatial expression patterns of *MtCEP1*, *2*, *4*, *5*, *6*, *8*, and *12* in roots inoculated with rhizobia for 0, 1, or 2 d.

Fig. S5. Mutation types at the first CRISPR/Cas9 editing target site of the *STF* locus.

Fig. S6. Single or double mutations of *MtCEP* genes had no significant influence on root development.

Fig. S7. Single or double mutations of *MtCEP* genes had no significant influence on nodulation.

Fig. S8. Relative expression levels of *MtCEP4*, *5*, *6*, *7*, *8*, *9*, *10*, *11*, and *13* in *Mtcep1*/*2*/*12C* and *Mtcep1*/*2*/*5*/*8*/*12C* under nitrogen starvation conditions.

Table S1. List of primers used in this study.

Table S2. The target sites selected for *STF* and *MtCEP* editing, and the corresponding primers used in our study.

Table S3. The sequences of the precursor proteins of the 16 MtCEPs identified from the R108 and Jemalong A17 Mt5.0 genome assemblies.

Table S4. Sequence of the three *MtU6* promoters.

Table S5. Mutation types of the CRISPR/Cas9 editing target sites of *MtCEP1*, *2*, and *12* in *Mtcep1/2/12C* and *Mtcep1/2/5/8/12C*.

erab093_suppl_Supplementary_Figures

erab093_suppl_Supplementary_Tables

## Data Availability

All data supporting the findings of this study are available within the paper and within its supplementary data published online. The multigene editing toolkit, and the stable mutants and transgenic GUS reporter lines used in this study are available from the corresponding author Tao Wang upon request.
