# Supplementary material for: Multigene editing reveals that MtCEP1/2/12 redundantly control lateral root and nodule number in Medicago truncatula
Source: J Exp Bot. 2021 Feb 26;72(10):3661–76. doi: 10.1093/jxb/erab093 (PMC8096600; doi:10.1093/jxb/erab093)
Supplement: erab093_suppl_Supplementary_Figures [file erab093_suppl_supplementary_figures.pdf]

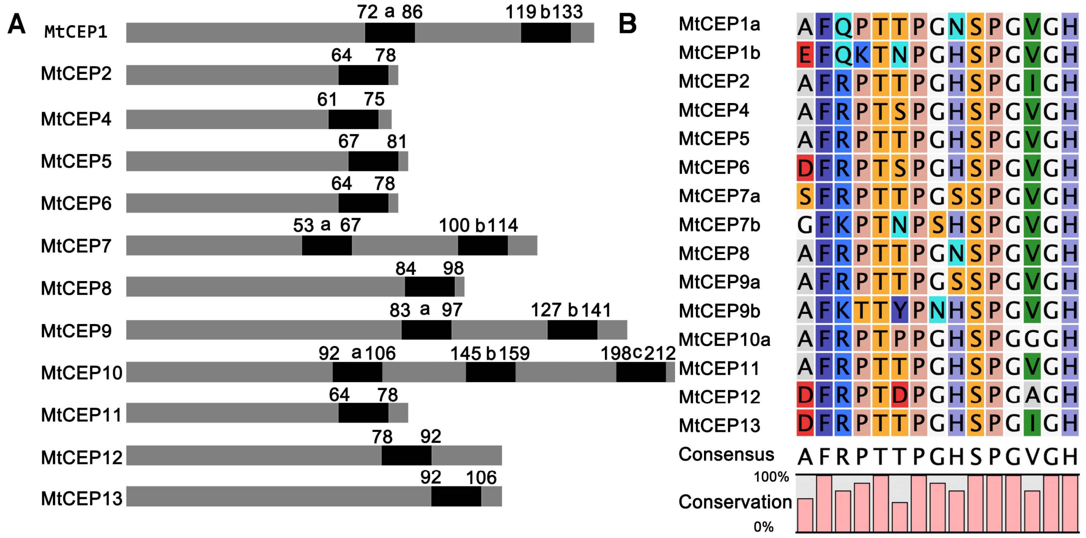

Supplementary Fig. S1. Representative scheme of the precursor protein and sequence alignment of CEP domains of the group I CEPs in R108. (A) The black regions represent the 15-amino-acid CEP domains, and the number indicates the location of the CEP domain in the precursor protein. The "a", "b" and "c" represent the CEP domain. (B) Sequence alignment of the 15-amino-acid CEP domains encoded by the 12 *CEP* genes in R108. The alignment was performed using CLC Sequence Viewer software. The 15 most highly conserved amino acid sequences are listed below, and the histogram shows the percentage of the probability for each of the amino acids.

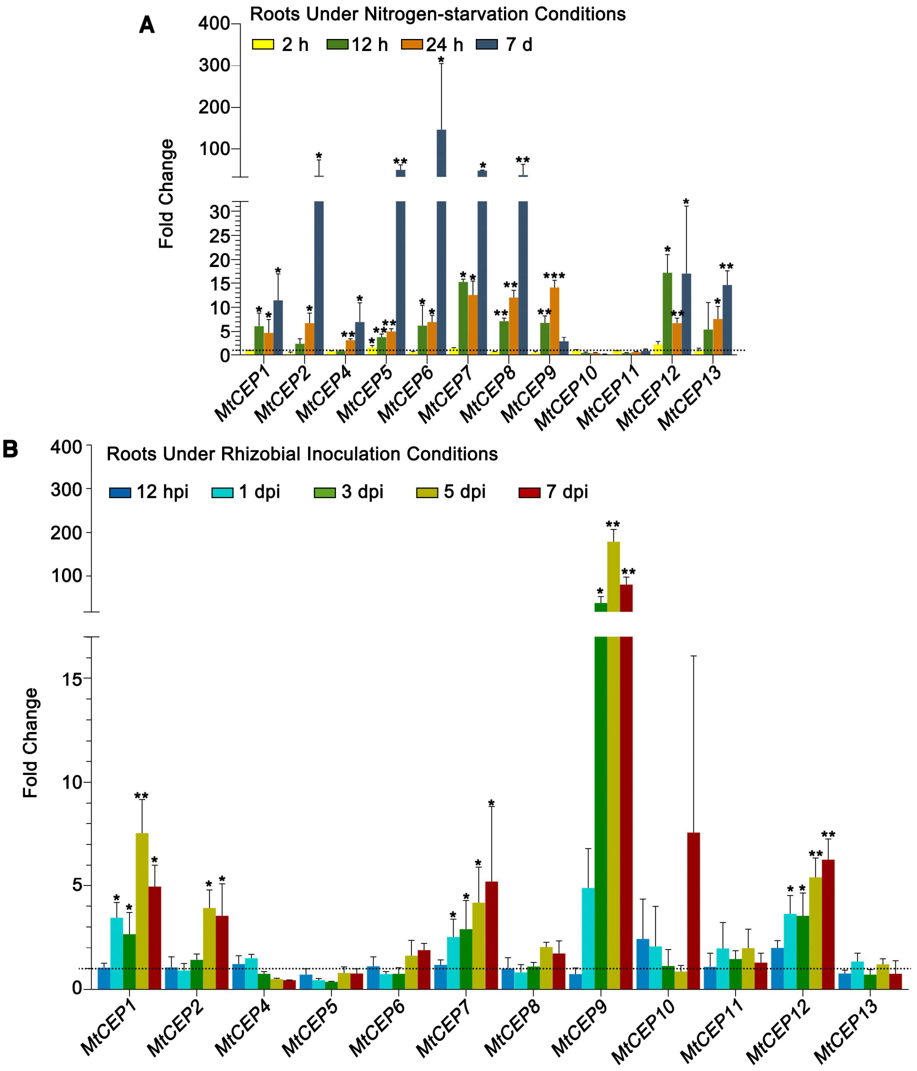

Supplementary Fig. S2. Fold change in the relative expression of group I *Mt*C*EP* genes under nitrogen-starvation conditions or in response to rhizobial inoculation. The germinated R108 seedlings were grown on Fåhraeus medium plates with 10 mM NO_3^- for 5 days and then transferred to Fåhraeus medium without nitrogen, and (A) roots were harvested separately after 0, 2, 12, and 24 hours (h) and 7 days (d). The data represented fold change of the roots under nitrogen starvation relative to control roots (0 h). (B) The remaining 12-day-old plants were inoculated with rhizobia, and the whole roots contained the primordium or nodules at 12 h post inoculation (hpi) and 1, 3, 5, and 7 days post inoculation (dpi). The data represented fold change of the inoculated roots relative to control roots (the plants under nitrogen starvation for 7 days). All the data were derived from three independent pools of roots and are presented as the means ± SDs. To highlight fold changes, the dotted line corresponds to a ratio of 1. The significant differences between treated and control conditions were determined with a paired two-tailed Student's *t*-test (*$p<0.05$, **$p<0.01$, ***$p<0.001$).

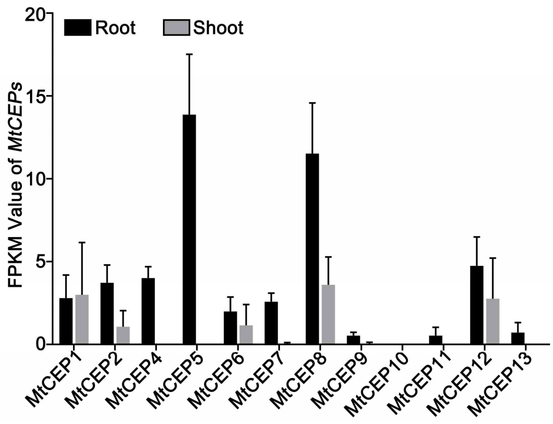

Supplementary Fig. S3. Expression of *MtCEP* genes in roots and shoots of R108 determined by RNA-seq. Expression data of the group I *MtCEP* genes; the data represent average values of fragments per kilobase of transcript per million mapped reads (FPKM) in the RNA-seq data of R108 at 5 dpi (Zhu et al., 2020). The data are the means of three different pools of materials ±SDs.

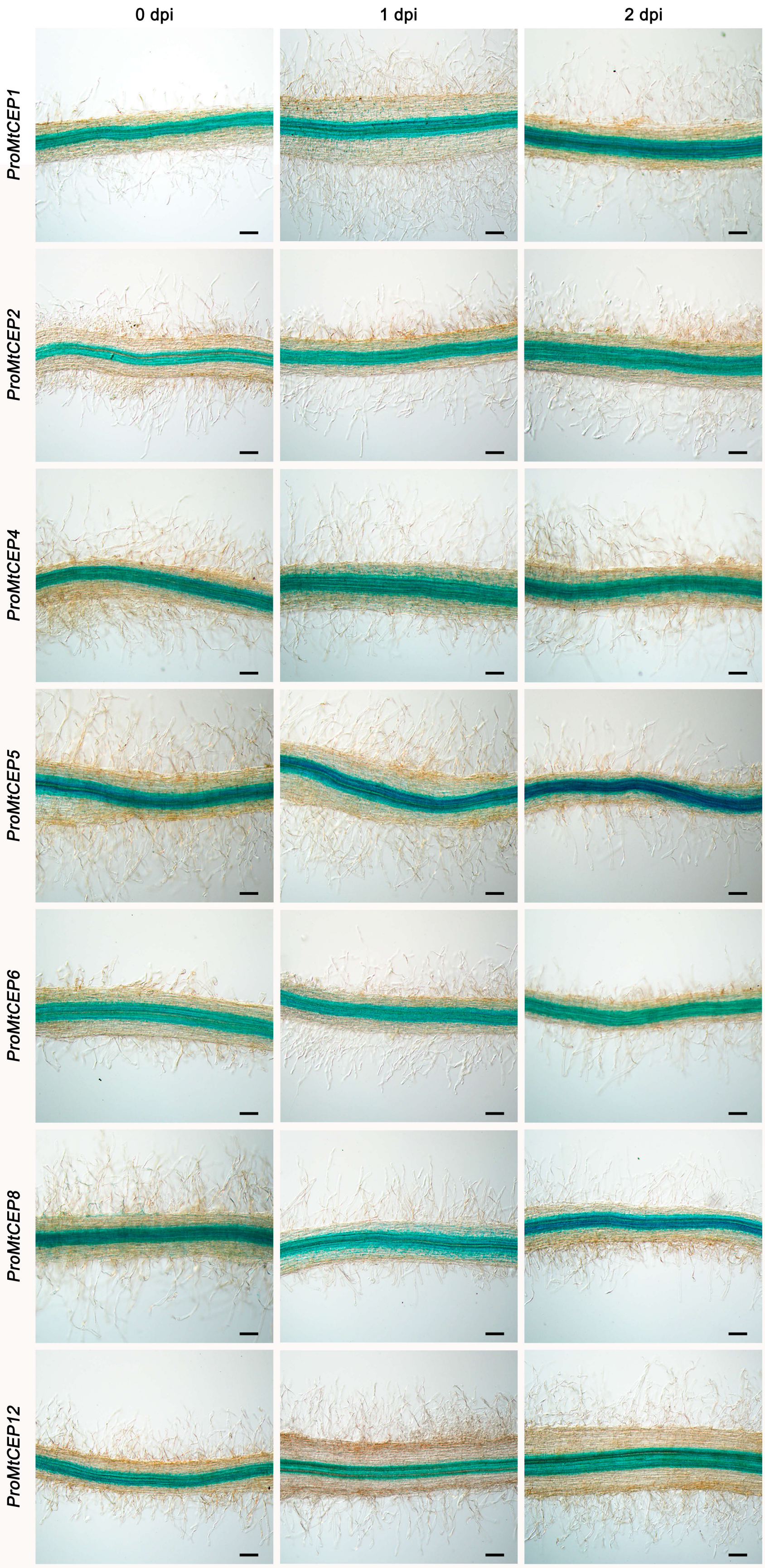

Supplementary Fig. S4. Spatial expression patterns of *MtCEP1*, *2*, *4*, *5*, *6*, *8* and *12* in roots inoculated with rhizobia for 0, 1 or 2 days. The stable transgenic plants

ProMtCEP1:*GUS*, *ProMtCEP2*:*GUS*, *ProMtCEP4*:*GUS*, *ProMtCEP5*:*GUS*, *ProMtCEP6*:*GUS*, *ProMtCEP8*:*GUS* and *ProMtCEP12*:*GUS* were germinated and grown in a soil/vermiculite mixture with Fåhraeus medium and inoculated with rhizobia 7 days later. The GUS staining images of roots (about 1 cm from the primary root tip). The bar represents 200 μm.

```
WT       TACAGCAAAGAAGAAACTTTATGGAAAGGAATGCC.ACGTGGCATATGATGCAGTTAACTTCTTCTTGTCCTACAGCT
stf-1-2  TACAGCAAA...................................................CGCTTTTATCCTACAGCT −50
stf-1-4  TACAGCAAAGAAGAAACTTTATGG..................CATATGATGCAGTTAACTTCTTCTTGTCCTACAGCT −17
stf-2-3  TACAGCAAAGAAGAAACTTTATGGAAAGGAA.....ACGTGGCATATGATGCAGTTAACTTCTTCTTGTCCTACAGCT −4
stf-2-5  TACAGCAAAGAAGAAACTTTATGG............................................TCCTACAGCT −43
stf-3-1  TACAGC.......................................................................T −70
stf-3-6  TACAGC.......................................................................c −70
stf-4-3  TAC...................................GTGGCATATGATGCAGTTAACTTCTTCTTGTCCTACAGCT −34
stf-4-4  TAC.......................................................................AGCT −70
stf-5-2  TACAGC.......................................................................T −70
stf-5-3  TACAGCAAAGAAGAAACTTTATGGAAAGGAATGCCCACGTGGCATATGATGCAGTTAACTTCTTCTTGTCCTACAGCT +1
stf-6-1  TACAGCAAAGAAGAAACTTTATGGAAAGGAATGC..ACGTGGCATATGATGtAGTTAACTTCTTCTTGTCCTACAGCT −1
stf-6-3  TACAGCAAAGAAGAAACTTTATGGAAAGGAATG...ACGTGGCATATGATGCAGTTAACTTCTTCTTGTCCTACAGCT −2
stf-7-1  TACAGCAAAGAAGAAACTTTATGGAAAGGAATGC..ACGTGGCATATGATGCAGTTAACTTCTTCTTGTCCTACAGCT −1
stf-7-4  TACAGCAAAGAAGAAACTTTATGGAAAGGA......ACGTGGCATATGATGCAGTTAACTTCTTCTTGTCCTACAGCT −5
stf-8-1  TACAGCAAAGAAGAAACTTTATGGAAAGGAATGCC..CGTGGCATATGATGCAGTTAACTTCTTCTTGTCCTACAGCT −1
stf-8-2  TACAGCAAAGAAGAAACTTTATGGAAAGGAATGCC..CGTGGCATATGATGCAGTTAACTTCTTCTTGTCCTACAGCc −1
stf-9-2  TACAGCAAAGAAGAAACTTTATGGAAAGGAATGC..ACGTGGCATATGATGCAGTTAACTTCTTCTTGTCCTACAGCT −1
stf-9-6  TACAGCAAAGAAGAAACTTTATGGAAAGGA......ACGTGGCATATGATGCAGTTAACTTCTTCTTGTCCTACAGCT −5
stf-10-1 TACAGCAAAGAAGAAACTTTATGGAAAGGAATGC..ACGTGGCATATGATGCAGTTAACTTCTTCTTGTCCTACAGCT −1
stf-10-2 TACAGCAAAGAAGAAACTTTATGGAAAGGA......ACGTGGCATATGATGCAGTTAACTTCTTCTTGTCCTACAGCT −5
stf-11-1 TACAGCAAAGAAGAAACTTTATGGAAAGGAATGC..ACGTGGCATATGATGCAGTTAACTTCTTCTTGTCCTACAGCT −1
stf-11-2 TACAGCAAAGAAGAAACTTTATGGAAAGGA......ACGTGGCATATGATGCAGTTAACTTCTTCTTGTCCTACAGCT −5
stf-12-1 TACAGCAAAGAAGAAACTTTATGGAAAGGAATGC..ACGTGGCATATGATGCAGTTAACTTCTTCTTGTCCTACAGCT −1
stf-12-2 TACAGCAAAGAAGAAACTTTATGGAAAGGA......ACGTGGCATATGATGCAGTTAACTTCTTCTTGTCCTACAGCT −5
stf-13-1 TACAGCAAAGAAGAAACTTTATGGAAAGGAATGCC..CGTGGCATATGATGCAGTTAACTTCTTCTTGTCCTACAGCT −1
stf-13-2 TACAGCAAAGAAGAAACTTTATGGAAAGGAATGC..ACGTGGCATATGATGCAGTTAACTTCTTCTTGTCCTACAGCT −1
stf-13-3 TACAGCAAAGAAGAAACTTTATGGAAAGGAAT...CACGTGGCATATGATGCAGTTAACTTCTTCTTGTCCTACAGCT −2
stf-14-2 TACAGC.......................................................................T −70
stf-14-3 TACAGCAAAGAAGAAACTTTATGGAAAGGAATGCCCACGTGGCATATGATGCAGTTAACTTCTTCTTGTCCTACAGCT +1
stf-15-2 TAC.......................................................................AGCT −70
stf-15-3 TAC...................................GTGGCATATGATGCAGTTAACTTCTTCTTGTCCTACAGCT −34
stf-16-5 TAC...................................GTGGCATATGATGCAGTTAACTTCTTCTTGTCCTACAGCT −70
stf-16-6 TAC.......................................................................AGCT −34
stf-17-2 TACAGCAAAGAAGAAACTTTATGG............................................TCCTACAGCT −43
stf-17-3 TACAGCAAAGAAGAAACTTTATGGAAAGGAA.....ACGTGGCATATGATGCAGTTAACTTCTTCTTGTCCTACAGCT −4
stf-18-3 TACAGCAAAGAAGAAACTTTATGGAAAG.........CGTGGCATATGATGCAGTTAACTTCTTCTTGTCCTACAGCT −8
stf-18-6 TACAGCAAAGAAGAAACTTTATGGAAAGGA.....CACGTGGCATATGATGCAGTTAACTTCTTCTTGTCCTACAGCT −4
stf-19-4 TACAGCAAAGAAGAAACTTTATGGAAAG.........CGTGGCATATGATGCAGTTAACTTCTTCTTGTCCTACAGCT −8
stf-19-6 TACAGCAAAGAAGAAACTTTATGG..................CATATGATGCAGTTAACTTCTTCTTGTCCTACAGCT −17
stf-20-1 TACAGCAAAGAAGAAACTTTATGGAAAG.........CGTGGCATATGATGCAGTTAACTTCTTCTTGTCCTACAGCT −8
stf-20-2 TACAGCAAAGAAGAAACTTTATGGAAAGGA.....CACGTGGCATATGATGCAGTTAACTTCTTCTTGTCCTACAGCT −4
stf-21-2 TAC.......................................................................AGCT −70
stf-21-3 TAC...................................GTGGCATATGATGCAGTTAACTTCTTCTTGTCCTACAGCT −34
stf-22-2 TACAGCAAAGAAGAAACTTTATGGAAAG........ACGTGGCATATGATGCAGTTAACTTCTTCTTGTCCTACAGCT −7
stf-22-3 TACAGCAAAGAAGAAACTTTATGGAAAGGAATGC..ACGTGGCATATGATGCAGTTAACTTCTTCTTGTCCTACAGCT −1
```

Supplementary Fig. S5. Mutation types at the first CRISPR/Cas9 editing target site of the *STF* locus. DNA fragments flanking Target 1/Target 2 at the *STF* locus were amplified from 22 transgenic lines regenerated from transformation of the original CRISPR/Cas9 plasmid targeting and ligated into PLB. Positive clones were sequenced. Sequences in the blue box were the first targets designed.

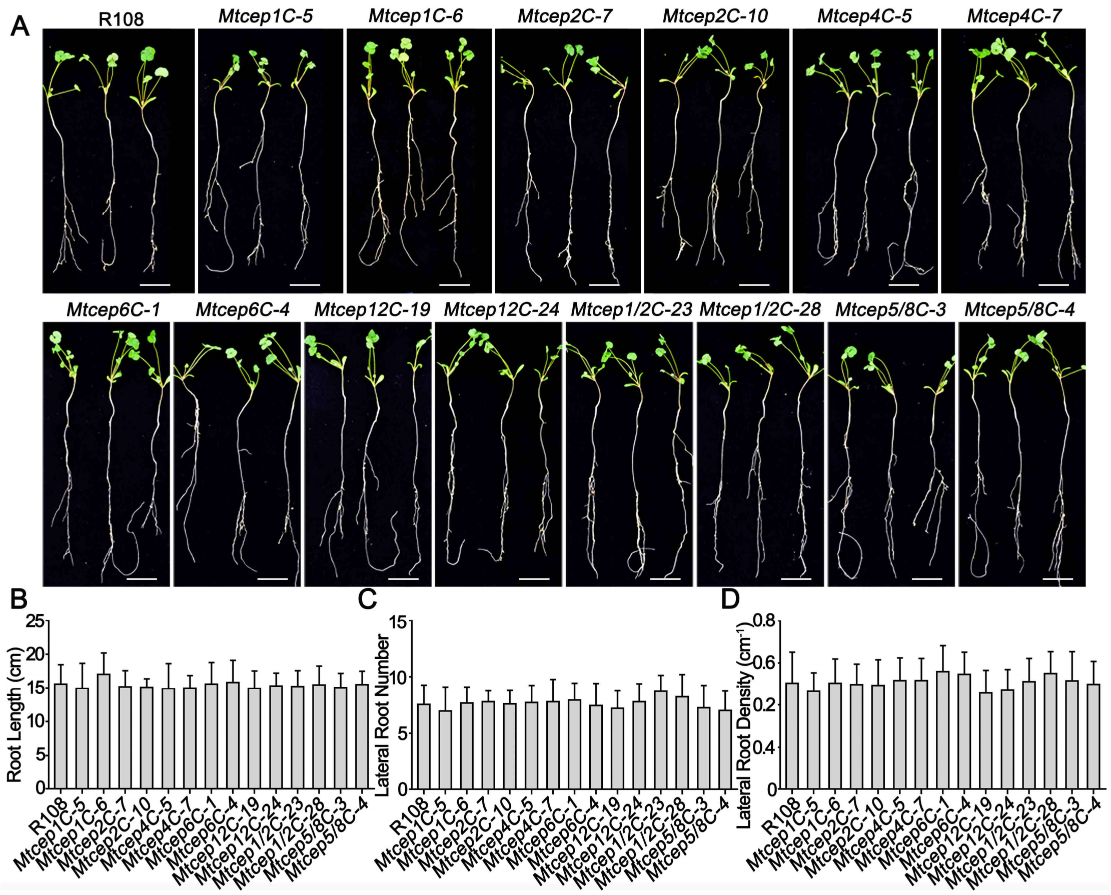

Supplementary Fig. S6. Single or double mutations of *MtCEP* genes had no significant influence on root development. Root growth phenotype of R108, *Mtcep1C*, *Mtcep2C*, *Mtcep4C*, *Mtcep6C*, *Mtcep12C, Mtcep1/2C* and *Mtcep5/8C* inoculated with *S. meliloti* Sm1021 after 1 week of growth in a soil/vermiculite mixture with N-free Fåhraeus medium. (A) Representative images of whole plants at 14 dpi and quantification of root length (B), lateral root number (C) and lateral root density (D). Similar results were obtained in three independent experiments; data are the means±SDs from one representative experiment. The significant differences were determined by ANOVA with a post hoc LSD test ($P < 0.05$, n > 15). The bar represents 2.5 cm.

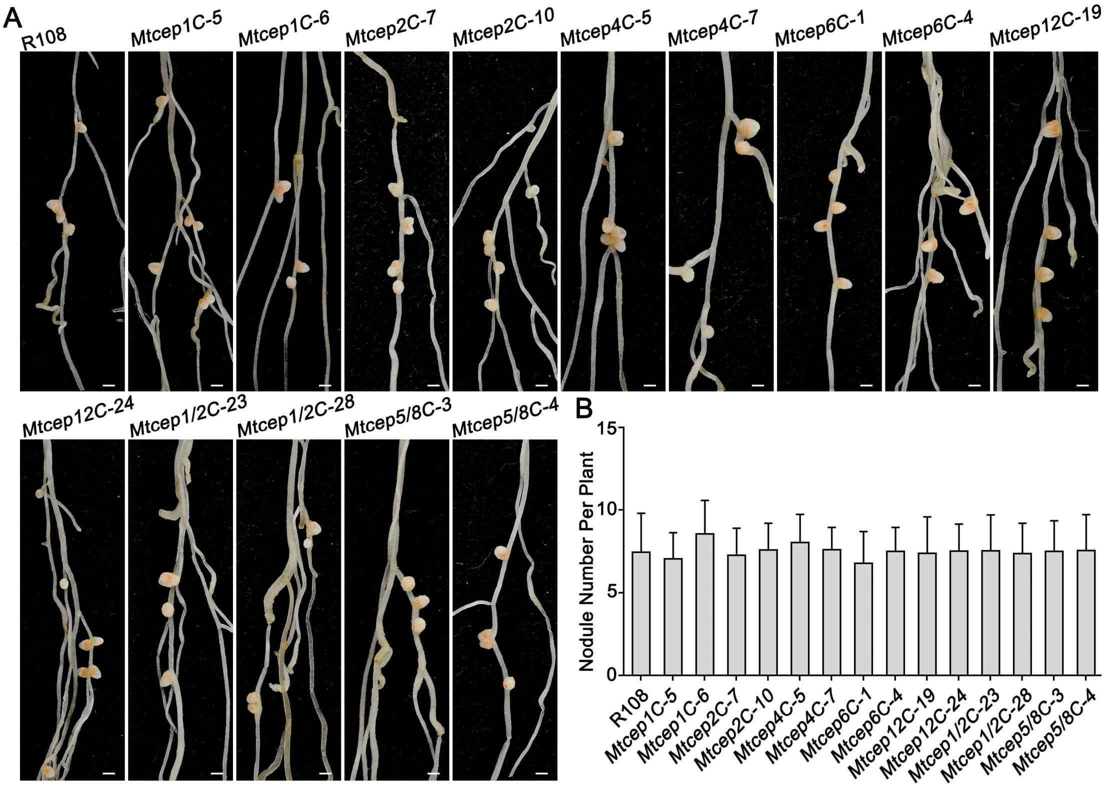

Supplementary Fig. S7. Single or double mutations of *MtCEP* genes had no significant influence on nodulation. Nodulation phenotype of R108, *Mtcep1/2/12C* and *Mtcep1/2/5/8/12C* at 14 dpi. The germinated seedlings were grown in a soil/vermiculite mixture with N-free Fåhraeus medium for 1 week and then inoculated with *S. meliloti* Sm1021. (A) Representative images of the nodulation phenotype at 14 dpi and (B) quantification of the nodules number at 14 dpi. Similar results were obtained in three independent experiments; data are the means±SDs from one representative experiment. The significant differences were determined by ANOVA with a post hoc LSD test ($P < 0.05$, $n > 15$). The bar represents 1 mm.

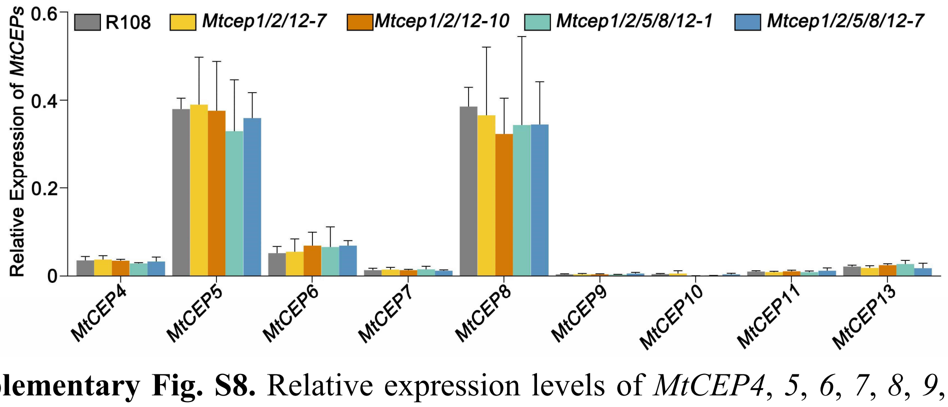

Supplementary Fig. S8. Relative expression levels of *MtCEP4*, *5*, *6*, *7*, *8*, *9*, *10*, *11* and 13 in *Mtcep1/2/12C* and *Mtcep1/2/5/8/12C* under nitrogen starvation conditions. The germinated R108, *Mtcep1/2/12C* and *Mtcep1/2/5/8/12C* seedlings were grown in liquid medium without nitrogen without N for 7 days, and then three different pools of roots were harvested separately. The data were derived from three independent pools of roots and were presented as the means±SDs. The significant differences between these mutants and R108 were determined with a paired two-tailed Student's *t*-test ($p<0.05$).
